# Qualitative Assessment of a Novel Intervention to Reduce Hospital Readmission Risk Among People with Diabetes

**DOI:** 10.18103/mra.v12i12.5882

**Published:** 2024-12

**Authors:** Samuel Tanner, Emily Brzana, Andrew Deak, Dominic Recco, Madeline Tivon, Felicia Dillard, Samantha Watts, Neil Kondamuri, Sarah B. Bass, Daniel J. Rubin

**Affiliations:** 1 Lewis Katz School of Medicine at Temple University, 3500 North Broad Street, Philadelphia, Pennsylvania 19140; 2 Lewis Katz School of Medicine at Temple University, Section of Endocrinology, Diabetes, and Metabolism, 3500 North Broad Street, Philadelphia, Pennsylvania 19140; 3 Temple University College of Public Health, Department of Social and Behavioral Sciences; 1301 Cecil B. Moore Ave., Philadelphia, PA 19122

**Keywords:** Diabetes mellitus, readmissions, qualitative methodology, thematic analysis, hospital discharge

## Abstract

**Purpose:**

To qualitatively assess a novel intervention, the Diabetes Transition of Hospital Care (DiaTOHC) Program, designed to reduce hospital readmissions within 30 days of discharge among people with diabetes.

**Methods:**

In a separately reported randomized controlled trial of the DiaTOHC intervention, hospitalized people with diabetes were identified as high risk for 30-day hospital readmission using the Diabetes Early Readmission Risk Indicator (DERRI^®^). Of these, 58 participants were randomized to the intervention. After the 30-day intervention, participants and study staff completed semi-structured interviews until saturation was achieved, yielding 21 participant and 4 staff interviews. Each one underwent thematic analysis.

**Results:**

Four themes were identified: (1) Participants were motivated to make lifestyle changes, (2) Weekly Navigator phone calls were an effective method to support participants, (3) The intervention improved some diabetes knowledge domains but not others, and (4) Perceived lack of control was associated with readmission. Participants with baseline hemoglobin A1C (A1C) ≥8% made more changes to their diabetes management due to the intervention but were less likely to review the educational materials and had more extreme blood glucose levels. Participants who completed fewer post-discharge phone calls were more likely to find the educational booklet helpful than those who completed more calls.

**Conclusions:**

Education, care coordination, and follow up are key components of the DiaTOHC Program that may improve diabetes self-management after a hospitalization and reduce readmission risk.

## Introduction

Early hospital readmission (i.e., within 30 days of discharge) is a widely used healthcare quality indicator and driver of cost.^1,2^ Therefore, the development and implementation of strategies to reduce 30-day readmission risk for people with diabetes is critical. Worldwide, the prevalence of diabetes in adults was estimated to be 10.5% (536.6 million people) in 2021, with a projected increase to 12.2% (783.2 million) in 2045.^3^ People with diabetes are more likely than those without diabetes to be readmitted,^4^ with readmission rates as high as 20.4%.^5^ Given these alarming numbers, interventions to reduce the risk of 30-day readmission in people with diabetes are urgently needed. However, there has been limited research on the effectiveness of such interventions in this vulnerable population.^6–11^

The Diabetes Transition of Hospital Care (DiaTOHC) Pilot Study was a non-blinded, randomized controlled trial of a novel intervention designed to reduce readmission risk of people with diabetes.^12^ This study identified people with diabetes who were at high risk for readmission using the Diabetes Early Readmission Risk Indicator (DERRI^®^).^5^ The DERRI is a model that predicts 30-day readmission risk based on 10 items, including employment status, preadmission insulin use, the number of macrovascular complications, laboratory results on admission, and recent prior hospital discharge. Participants were randomized to either a novel intervention designed to reduce readmission risk (DiaTOHC) or usual care. The DiaTOHC intervention, described in more detail below, consisted of diabetes therapy adjustment upon discharge by an endocrinologist, education, and post-discharge support. This pilot trial showed a non-significant but measurable reduction in 30-day readmission risk among participants with a baseline A1C level greater than 7%.

In addition to the quantitative analysis, a qualitative substudy was planned to investigate the perceptions of DiaTOHC participants about the intervention to better understand their experience and the effects of the intervention. Given that interventions designed to reduce readmission risk have had variable results in people with diabetes,^11^ this qualitative substudy was conducted to inform potential improvements to interventions like the DiaTOHC program. Furthermore, prior qualitative studies have provided important insights into drivers of readmission risk, such as poor health literacy, health systems failures, and social determinants of health.^13,14^ We are unaware, however, of previously published qualitative analyses of readmission interventions in people with diabetes.

## Methods

### STUDY DESIGN, PROCEDURES, AND ETHICS

This was a qualitative study of semi-structured interviews. At 5 to 12 weeks after hospital discharge, DiaTOHC intervention participants were interviewed using a guide consisting of 20 questions (Table 1). The questions evaluated knowledge of the A1C test, thoughts regarding the diabetes education material, changes to diabetes management, opinion of Navigator follow-up, and experience following discharge instructions. These questions were developed by the project lead in collaboration with the study team. They were designed to explore the potential effects of the Intervention from participants’ perspectives and invite feedback on the program. Each participant interview was conducted in-person by one of the Research Coordinators, digitally recorded, and transcribed by a study team member. Participants were given $50 USD for providing an interview. Interviews were conducted on sequentially enrolled participants until thematic saturation occurred (i.e., an additional interview would not contribute new information).^15^

In addition to participants, two study team Navigators and two research coordinators were interviewed by the principal investigator. Including study team members enabled qualitative analyses of multiple perspectives. Topics included the efficacy of the educational materials, perceived strengths and weaknesses of the intervention, barriers to delivering the intervention, and opportunities to improve its design.

The protocol was approved by the Temple University Institutional Review Board (#24306). Participants provided written informed consent to participate.

### PARTICIPANTS AND SETTING

This study was conducted at Temple University Hospital, an urban, academic medical center in Philadelphia, PA. In the parent DiaTOHC Pilot Study, participants were randomized to the DiaTOHC Intervention or Usual Care. After completing the 30-day post-discharge follow-up period, only participants randomized to the Intervention group were invited to interview for the qualitative study presented here. Included patients had an established diagnosis of diabetes (preadmission use of a diabetes-specific medication and/or documentation of the diagnosis), age greater than 18 years, high predicted risk of 30-day readmission (≥27%) based on the DERRI^®,5^ hospital admission to a non-critical care unit and participated in at least 1 post-discharge phone call. People were excluded for pregnancy, binge drinking (at least 5 alcoholic drinks for males or 4 alcoholic drinks for females on the same day), drug abuse within 3 months before admission, receiving palliative care during the hospitalization, participation in another readmission risk reduction program, planned or actual transfer to another hospital or subacute facility, discharge expected within 12 hours, lack of access to a phone, living more than 30 miles away from the hospital, A1C <5.7% (39 mmol/mol), and inability to speak English. After enrollment, subjects were excluded upon transfer to another hospital or subacute facility, discharge to hospice or a long-term care facility, signing out against medical advice, or inpatient death. The DERRI^®^ is a publicly available tool that predicts the risk of readmission based on 10 factors including laboratory values, diabetic complications, use of insulin, home zip code, employment status, and prior hospitalization.^5^

### DiaTOHC INTERVENTION

Briefly, the Intervention consisted of diabetes therapy adjustment upon hospital discharge, education, and post-discharge support. Diabetes therapy upon discharge was determined by a study endocrinologist using an A1C-based algorithm based on previously published work and American Diabetes Association (ADA) guidelines.^16,17^ DiaTOHC education consisted of two parts delivered by a study team Navigator over the phone before discharge or 1 to 3 days after discharge according to participant availability. Notably, the Navigators had no specialized diabetes training. The first part was diabetes discharge instructions and education using a 19-page booklet designed for the study based on ADA guidelines that includes information on nutrition, physical activity, and self-care survival skills, such as how to recognize and treat hypoglycemia and hyperglycemia,^18^ as well as instructions on using diabetes medications.^19^ Additionally, participants who had not completed a formal diabetes education program in the prior 12 months were referred to the Temple Diabetes Center, an ADA-certified outpatient diabetes education center. For the second part of education, a Navigator reviewed the discharge plan with participants, covering the treatment plan, how to take medications, reasons for and importance of follow-up appointments and testing, and how to reach post-hospital providers.

For post-discharge support, a Navigator placed up to 4 additional phone calls weekly during the 30 days following hospital discharge. On these calls, the Navigator would reinforce the education and review blood glucose levels. If reported blood glucose levels were <70 or >240 mg/dL (3.9 or 13.3 mmol/L), then the Navigator notified a study physician, who contacted the subject by phone to adjust diabetes therapy per protocol.^12^ In addition, intervention subjects received a referral fora nursing visit in the home to assess medical needs for support at home. Barriers to following the discharge plan, such as transportation, food, housing, and financial issues, were assessed, and Navigators connected participants with community resources as needed. More DiaTOHC program details are available elsewhere.^12^

### ANALYSIS

Two investigators conducted inductive thematic analysis of interview transcripts.^20^ Each investigator coded each transcript independently, then compared codes to achieve consensus. Discordant codes were resolved by the principal investigator. After applying unique codes to the first 10 interviews, all codes were compiled under their corresponding question, and a standardized coding language was developed. These generalized codes were applied to the remaining interviews, and additional unique codes were applied as needed to capture new information. Following initial coding, all standardized codes were imported into the web-based software Dedoose (Manhattan Beach, CA), and transcriptions were coded using the generalized codes.

Descriptors categorizing participants by admission A1C (≥8% or <8%) and number of Navigator follow up phone calls (0 to 2 or 3 to 5) were applied to each interview to allow for stratification on these factors. Frequency of code application was assessed to identify key ideas.^21^

## Results

A total of 21 participants who received the DiaTOHC intervention were interviewed (Table 2). The median age was 58 (range, 32 – 77 years), 52% were female, and 90% had Type 2 diabetes. The median A1C was 8.8% (range, 6.0 – 15.5%). All 21 participants had been discharged from a hospital in the 90 days prior to the index admission.

**Figure 1.**
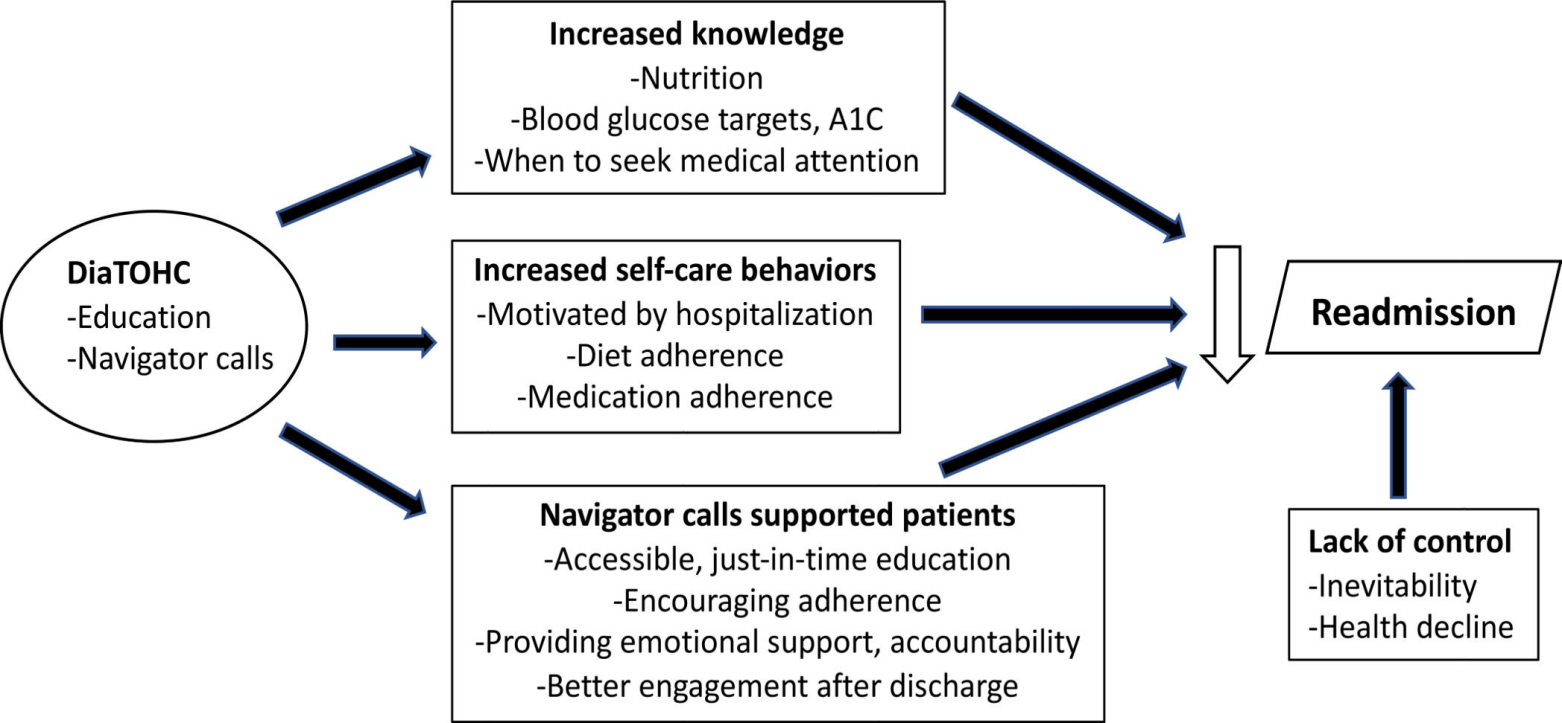
Themes and subthemes from interviews. Arrows represent association. DiaTOHC: Diabetes Transition of Hospital Care Program

We identified four main themes: 1) Participants improved self-care behaviors 2) Navigator phone calls supported participants, 3) The intervention improved some diabetes knowledge domains, and 4) Perceived lack of control was associated with readmission (Figure 1).

### PARTICIPANTS IMPROVED SELF-CARE BEHAVIORS

Most participants in the intervention improved at least one self-care behavior, such as dietary and medication adherence, after hospital discharge. One participant said, *“if you don’t change your eating habits, you might as well forget about trying to do anything else.*” Diet changes often included reducing intake of high carbohydrate foods (e.g., baked goods, cereal, pizza), eating smaller portions, and/or increasing fruit and vegetable intake. Many attributed these improvements to the diabetes education material, with one participant saying that the program *“was a good incentive to get me on track...My sister can’t believe I gave up the juice”* and another noting that *“my eating habits, they’re much better. I’m more aware.”*

In addition to dietary changes, 38% of participants stated they improved their medication adherence due to the intervention. One said, *“I just took some of [my medication]. I never took all of it. But now I have them labeled, and I know what to take and how much to take.”* Upon being asked if she improved her insulin adherence, another participant responded, *“Oh, I wasn’t taking it at all..., but now I am. And I do see a difference.”*

Some participants viewed their hospitalization as a wakeup call to take better care of their diabetes. When one was asked how the intervention could be improved, he responded, *“I mean you can tell ‘em, you can lead a horse to water, he don’t drink it. Know what I’m sayin? I was that same way.”*

Interviewer: What changed for you?

Patient: Surgery, getting tired of having to go through surgery....

Interviewer: So, you were ready to hear it?

Patient: Yep.

Another participant stated that her hospitalization made her *“take a look at what part I was playing in all this. I had to, you know, take the responsibility for it.”*

### NAVIGATOR PHONE CALLS SUPPORTED PARTICIPANTS

Participants were asked about the diabetes education they received during their hospitalization, outpatient diabetes classes, and the weekly Navigator phone calls. Most participants reported the education they received prior to discharge was helpful but could not provide details on what they had learned. Most participants reported that they did not have questions about diabetes at the time of discharge. The Navigators reported that the inpatient education sessions were subject to frequent interruptions by providers, visitors, and procedures. In addition, the Navigators reported that they had difficulty engaging participants in diabetes education while they were hospitalized. As one Navigator stated, *“[The patients] are still sick when they leave ... they feel terrible, and they just want to go home and get some sleep. There is only so much you can teach.”* In contrast, the Navigators observed that participants were generally more engaged on phone calls completed at home.

Another perceived benefit of the phone calls was just-in-time education. One participant said, *“They are right on the phone, and you can bring it up. When you normally get to the doctor’s office a month later you forget.”* Participants also found the ease with which they could contact a Navigator helpful, with one saying, *“If the [home] nurse left and I needed to ask a question or I wasn’t feeling good, I could tell [the Navigator] and she was right there.”*

In addition to education, Navigators offered more general support and encouragement. Participants reported that the Navigators encouraged adherence to their diabetes self-management plan, in part by providing accountability. One participant commented that the Navigator *“[made] sure I stayed on top of getting my sugar tested and [made] sure I was eating right.”* Exemplifying the emotional support offered, one participant said, *“Sometimes I was down so [the study nurse] gave me some encouragement.”* Another said, *“You know [the Navigator], he was a good supporter of me.... If you get people who support you, that is when you do the right thing. Because I get tired of the doctor and my family telling me what I should and shouldn’t eat.”* All but one participant reported finding the Navigator phone calls helpful.

In contrast to education provided with the intervention, none of the interviewed participants attended outpatient diabetes classes as recommended. One participant expressed her concern about finding time to attend classes, saying, *“I can’t pile anything else on me and stay in control.”* Several participants stated that the diabetes education classes felt like a burden, so they prioritized doctor appointments. As one Navigator said, *“I just think their conditions are so complex and they are so overwhelmed. And a lot of times it will happen to them all at once. Because they will go 20 years without seeing a doctor and then all of the sudden, they have heart failure and diabetes, and ... now they are so overwhelmed.”* Some participants declined to engage in diabetes education classes because they felt like they already had a baseline of knowledge and instead needed more personalized support.

### THE INTERVENTION IMPROVED SOME DIABETES KNOWLEDGE DOMAINS

Interviews explored participants’ understanding of diabetes-related topics covered by the Navigators, specifically, diet and medication regimens, the symptoms of hyper- and hypo-glycemia, interpretation of the A1C test, and their personal A1C levels. Most participants reported that they were already familiar with the dietary information in the booklet, but that it was helpful as a reminder and tool to educate family members. One person said *“I already knew the portions, but it is a good reminder of exactly what you should do. And that is why I keep it with me so I can look at it every once in a while, ... it does help a lot.* “Nearly half the interviewees reported making dietary changes specifically based on information in the booklet.

When participants were asked what they found helpful in the booklet, the most cited section was the Diabetes Zones of “All Clear” (green, blood glucose levels in target range), “Stop and Call” (yellow, moderate hyperglycemia and/or hypoglycemia), and “Emergency” (red, severe hypoglycemia and/or hypoglycemia) (See Supplement). One participant found this color system a simple way to understand how well she was doing with diabetes management. She said, *“What Diabetes Zone are you in today? Green, yellow, or red. Every day check your blood sugar as directed, take your medicine, follow your diet, try to get some physical activity. Green zone all clear, this zone is your goal.”* Another participant judged the quality of her diabetes management in terms of the time she spent in the green zone, analogous to time in the target range. Although no participant specifically mentioned calling their provider when they were in the yellow zone, they did appreciate knowing when it was appropriate to seek non-emergency medical assistance.

Perhaps one of the largest gaps in patient knowledge was the A1C test. Prior to the intervention, only one participant (5%) knew the meaning of the A1C test. Following the intervention, 14 participants (67%) recalled that a Navigator addressed the test, and 10 (48%) of these participants accurately described its interpretation. However, only 5 participants (24%) correctly identified their A1C target, and 8 participants (38%) accurately reported their most recent A1C level.

### PERCEIVED LACK OF CONTROL WAS ASSOCIATED WITH READMISSION

The interviews explored whether participants who had a hospital readmission believed that it could have been prevented. Almost all these participants thought their readmission could not have been prevented, especially when it was a result of health deterioration. For example, one participant stated, *“Once your heart starts going bad, it can go on its own whenever it’s ready.”* Another said, *“It’s my heart. So, can’t control that.”* One participant expressed uncertainty regarding his surgery recovery, noting that *“you don’t know until you get out and start functioning what’s going to happen to you.”* These statements demonstrate the belief of some interviewees that hospital readmission was inevitable.

### DIFFERENCES IN PERCEPTION BASED ON ADMISSION A1C VALUE (STRATIFIED ANALYSIS)

Compared to participants with an admission A1C ≥8%, those with an A1C <8% were more likely to report that they found the study material consistent with prior diabetes education. This was reflected in their better understanding of the A1C test. The A1C <8% group also described taking a more active role in their self-care. In contrast, the A1C ≥8% group was less likely to independently review the provided education material and more likely to have difficulty adhering to their discharge instructions. They were also more likely, however, to recall discussing the discharge plan with the Navigator and making changes in diabetes self-management prompted by the intervention.

### DIFFERENCES IN PERCEPTION BASED ON QUANTITY OF NAVIGATOR FOLLOW-UP CALLS (STRATIFIED ANALYSIS)

Compared to participants who received between 3 and 5 Navigator phone calls, those who received less than 3 calls more frequently wanted additional education on diabetes management and medication. Furthermore, this cohort also felt that more time spent reviewing the discharge plan would have been beneficial. They were also less likely to recall the Navigator reviewing the education booklet. These participants, however, were more likely to find the booklet helpful and more likely to make dietary changes based on the nutritional education, such as decreasing intake of pizza and cereal. In contrast, the participants who received 3 to 5 calls were more likely to report a positive experience with the Navigator and felt that the Navigator covered everything necessary. While all participants reported making diet changes due to the intervention, only the cohort completing 3 to 5 phone calls reported improving their medication compliance and eating smaller portion sizes.

## Discussion

This qualitative assessment of a readmission risk reduction intervention among high-risk people with diabetes identified several important aspects of participants’ experience. Motivated by their hospitalization, many participants improved some self-care behaviors. This reinforces the concept that hospital discharge represents an important opportunity to modify diabetes management.^22,23^ Participants appreciated the phone calls from the Navigators, which provided just-in-time diabetes education, emotional support, accountability, triage to a physician for diabetes management, and encouraged adherence to the self-management plan. This suggests that care navigation is a critical component of the DiaTOHC Program, consistent with literature in other populations showing that transition of care navigation is usually associated with reduced readmission risk.^24–26^

Participants reported improvement in knowledge about nutrition, blood glucose targets, the A1C test, and when to seek medical attention. Despite these positive perceptions of the DiaTOHC intervention, most participants who experienced a readmission believed that it was inevitable, especially when due to a deterioration in health. The belief that hospital readmission is inevitable poses a barrier to reducing readmission risk. This has been previously described among people with diabetes.^27^ Such belief may be partly because many hospitalized people with diabetes have multiple co-morbidities, making management complex.

Both participants and Navigators found that post-hospitalization phone calls were an effective strategy for education. In contrast, Navigators observed that phone calls placed to participants while they were still in the hospital yielded less effective education. This may be attributable to the patient’s state of well-being while in the hospital and limitations in the ability to engage people by phone in this state. Patients experience a myriad of stressors during hospitalization including lack of sleep, disruption of circadian rhythms, challenging medical situations, poor nourishment, pain, medication changes that may affect cognition, and physical deconditioning.^28^ Nevertheless, in-person inpatient diabetes education has been independently associated with reduced 30-day readmissions.^7,29^ However, if patients are unwilling or unable to engage in education while in the hospital, there needs to be a process in place for them to receive education after discharge.

Part of the importance of the post-discharge Navigator phone calls appears to be related to providing convenient, individualized support as opposed to a standard, generic outpatient diabetes education program that required scheduling and transportation. It is telling that none of the participants attended outpatient diabetes education classes. The median duration of diabetes in this cohort was 14 years, so many participants had attended some form of outpatient diabetes education in the past and did not want to repeat that experience. Additionally, the post-hospitalization period was often a busy time for the participants with follow up appointments with their physicians. Thus, participants prioritized these appointments over outpatient diabetes classes. Navigator phone calls are critical to ensuring people receive the necessary diabetes follow-up education and support, which may aid in reducing diabetes distress and readmission risk.

The most appreciated component of diabetes education in the intervention was the Diabetes Zones, a novel, one-page set of color-coded guidance on managing high and low blood glucose levels symptoms. It appears that participants were eager for guidance on how to gauge their diabetes self-management and when to seek help. This appeared to be a more meaningful way of communicating the effectiveness of diabetes self-management than the A1C test. Many participants struggled to explain the significance of the A1C test, let alone recall what their current and goal A1C levels were. This is consistent with a previous qualitative study of people with diabetes who had experienced a readmission.^13^ Most patients refer to A1C as something their provider uses to understand how well they are managing their diabetes. Because it is only discussed during provider visits, A1C may have less influence on the perception of self-efficacy. Moreover, participants appreciated the specific guidance on when to contact a provider. Participants felt as though this gave them permission to contact the doctor to help them with diabetes management when otherwise they may have felt ashamed or bothersome. Interestingly, participants with an A1C ≥8% were more likely than participants with lower A1C levels to find the Diabetes Zones education helpful, suggesting that it may be more beneficial to those with higher blood glucose levels.

More engagement with the Navigators as measured by the number of completed post-discharge phone calls was associated with greater satisfaction with the program and more improvements in diabetes self-management behaviors. Not surprisingly, engagement with non-medical intervention is associated with improvements in both diabetes self-management and weight loss.^30,31^ What remains unclear from the present study is why the engagement of participants varied and how engagement in the DiaTOHC Program could be improved.

Participants’ experiences with the intervention and post-discharge period varied by baseline A1C level. Those with a higher baseline A1C level seemed to benefit more from the intervention in terms of diabetes knowledge and self-management behaviors but had more difficulty adhering to their discharge instructions. This suggests that the intervention could provide more benefit if participants with higher A1C levels receive more intense support than those with lower A1C levels. Of note, the intervention did not decrease A1C levels more than usual care at 3 months after discharge.^12^

There have been other studies of multifaceted interventions intended to improve the hospital discharge transition among people with diabetes^32^ and more general populations.^26,33,34^ Results of these trials have been mixed, with some studies showing benefit in terms of readmission risk reduction and others finding no benefit. Furthermore, it remains unclear which components of multicomponent interventions provide benefit. None of these other interventions have been examined with qualitative methods. The present study suggests that qualitative analysis may provide some insight into identifying key components of interventions and how to customize them to maximize effectiveness.

There are limitations to this study. First, this was a single-center study at an urban academic medical center and the study population was relatively homogenous, with most participants being Black, lower income, and disabled. However, this population tends to be at higher risk for readmission and may stand to benefit from the intervention than a lower risk population. Second, there is probably volunteer bias in patients choosing to enroll in the trial and remain in the study. Third, inferences about cause and effect are limited by the lack of interviews of control group participants. The main strength of this study is that it represents the first qualitative assessment of a hospital readmission risk reduction intervention among people with diabetes.

## Conclusions

In summary, the DiaTOHC intervention is acceptable to patients and appears to address some of the factors contributing to readmission risk such as health system failures and poor health literacy while improving self-management. The intervention’s combination of education, care coordination and follow up may augment medical management in reducing readmission risk in the post-hospitalization period. Additional research is needed to both confirm these themes and further optimize the intervention.
